# 3D Printing and Property of Biomimetic Hydroxyapatite Scaffold

**DOI:** 10.3390/biomimetics9110714

**Published:** 2024-11-20

**Authors:** Xueni Zhao, Lingna Li, Yu Zhang, Zhaoyang Liu, Haotian Xing, Zexin Gu

**Affiliations:** College of Mechanical and Electrical Engineering, Shaanxi University of Science and Technology, Xi’an 710021, China; 220511023@sust.edu.cn (L.L.);

**Keywords:** 3D printing, hydroxyapatite whisker, bioceramic scaffold, mechanical property

## Abstract

The 3D printing of a biomimetic scaffold with a high hydroxyapatite (HA) content (>80%) and excellent mechanical property is a serious challenge because of the difficulty of forming and printing, insufficient cohesion, and low mechanical property of the scaffold. In this study, hydroxyapatite whiskers (HAWs), with their superior mechanical property, biodegradability, and biocompatibility, were used to reinforce spherical HA scaffolds by 3D printing. The compressive strength and energy absorption capacity of HAW-reinforced spherical HA (HAW/HA) scaffolds increased when the HAW/HA ratio increased from 0:10 to 4:6 and then dropped with any further increases in the HAW/HA ratio. Bioceramic content (HAWs and spherical HA) in the scaffolds reached 83%, and the scaffold with a HAW/HA ratio of 4:6 (4-HAW/HA) exhibited an optimum compressive strength and energy absorption capacity. The scaffold using polyvinyl alcohol (PVA) as an additive possessed a good bonding between HA and PVA as well as a higher strength, which allowed the scaffold to bear a higher stress at the same strain. The compressive strength and toughness of the 4-HAW/HA-PVA scaffold were 1.96 and 1.63 times that of the 4-HAW/HA scaffold with hydroxypropyl methyl cellulose (HPMC), respectively. The mechanical property and inorganic components of the biomimetic HAW/HA scaffold were similar to those of human bone, which would make it ideal for repairing bone defects.

## 1. Introduction

Diseases such as trauma, tumor resection, and infection lead to large bone defects which become common problems in orthopedics [1,2]. Bioceramic composites demonstrate great potential in treating large bone defects (2 cm or a range of more than 1.5 times the diameter of long bones) that usually rely on bone graft transplantation in the human body [3]. Hydroxyapatite (HA), a well-known compound of the calcium phosphate family and the principal component of bone mineral, has some advantages such as biodegradability, biocompatibility, nontoxicity, and non-immunogenicity, and it has been used as a bone substitute for repairing bone defects [4,5]. However, HA has some limitations, such as low strength and toughness, which can be solved by introducing some reinforcements into pure HA [6].

As additive manufacturing, 3D printing can readily design and manufacture a scaffold with an internal structure, shape, porosity, and pore size [7]. HA scaffolds printed in 3D afford unparalleled probabilities in bone tissue engineering, which can produce various personalized implants with complex structures to treat critical-sized bone defects [8]. As well, even cell distribution and vascular integration on 3D and 4D printed HA scaffolds incorporated with biofunctionalities can be improved [9,10,11]. Wang et al. [12] successfully prepared biphasic calcium phosphate scaffolds with pore sizes between 300 μm and 1 mm through 3D printing, demonstrating a powerful ability to process complex structures with high resolution and precision. Zhang et al. prepared HA scaffolds at a nozzle speed ranging from 240 mm/min to 600 mm/min by inkjet 3D printing. The diameter of the nozzles was between 110 μm and 800 μm, the distance of the two filaments ranged from 500 μm to 1400 μm, and the thickness of the slice layers varied with the nozzle diameter, following an empirical formula (h=0.8 d±0.02 μm). The compressive strength of the scaffolds with different porosity (60%, 70%, and 80%) after sintering was 5.5 MPa, 3.2 MPa, and 0.9 MPa, respectively [13]. Although the mechanical performance of the printed HA scaffolds can be slightly improved after sintering, the printed scaffold demonstrated great shrinkage after sintering, which could result in some microcracks in the scaffold and degrade its mechanical properties. This greatly limits the application of HA scaffolds in treating bone defects.

HA whiskers have been applied as biocompatible reinforcements in biomaterials and have also been applied to orthopedic implant fixation, such as interbody spinal fusion [14]. The mechanical properties of polymer and ceramics can be significantly enhanced by introducing HA whiskers. Synthesized HA whiskers or spherical HA powder were introduced to reinforce high-density polyethylene (HDPE). The HA whiskers had a length of 18.0 ± 8.9 μm, a width of 2.3 ± 0.8 μm, and an aspect ratio of 7.9 ± 3.4. The HDPE composites reinforced with HA of various volumes (10, 20, 30, 40, 50, and 60 vol%) were prepared by two thermomechanical processing steps. The results showed that the HA whisker-reinforced HDPE had an improved elastic modulus (>10 GPa), tensile strength (−30 MPa), and failure work (~800 N·mm) compared with the spherical HA-reinforced composites [15]. Cubic or ellipsoidal NaCl was used as porogen to fabricate a biomaterial scaffold [16]. Conrad et al. used 20 vol% of HA whiskers as a reinforcement to prepare a HA scaffold. HA whisker-reinforced PEEK scaffolds were fabricated by an ellipsoidal NaCl porogen. The HAWs reinforced the porous polyetheretherketone (PEEK) scaffold with the addition of the 20 vol% of HA whiskers, and the compressive strength of the scaffold with 75–80 vol% porosity was only 1.0–2.2 MPa. The scaffolds were beneficial to cell infiltration and bone growth for implant fixation [17]. The compression strength of the porous nano HA whisker (nHA_W_)/β-tricalcium phosphate (β-TCP)/bioglass(BG) ceramic scaffolds increased compared with that of the pure β-TCP and β-TCP/BG scaffolds with the same porosity, indicating that the scaffolds were strengthened by the addition of nano HA whiskers with a length of −2 μm and a diameter of 100 nm [18]. Additionally, the scaffold surfaces were also modified by the HA whiskers, which improved the bioactivity of the scaffolds. HA whiskers were incorporated into the calcium silicate to enhance the strength of the scaffolds by selective laser sintering [19]. The compressive strength of the composites increased as the HA whisker content was reduced to less than 20 wt%; conversely, the compressive strength decreased by increasing the HA whisker content. The HA whiskers were about 500 nm long and 10 nm wide. The compressive strength increased from 18.19 ± 1.25 MPa to 27.28 ± 0.70 MPa when 20 wt% HA whiskers were added. However, the high sintering temperature of the calcium silicate ceramic scaffold may have an adverse impact on the biological property. Suchanek et al. [20] prepared hydroxyapatite whisker-reinforced hydroxyapatite (HA/HA whisker) composites with 10%, 20%, and 30% HA whiskers (20–50 nm in diameter and 100–300 nm in length) through pressureless sintering, hot pressing, and hot isostatic pressing. HA/HA whisker composites exhibited the best properties by hot isostatic pressing at 1000–1100 °C (2 h, 190 MPa) due to the reinforcing effect of HA whiskers in the HA matrix. Compared with the fracture toughness (1.0 MPa·m^1/2^) of the non-reinforced HA matrix, the relative densities and fracture toughness of the HA/HA whisker composites reached 97.0–99.5% and 1.4–2.0 MPa·m^1/2^, respectively.

Although HA whisker-reinforced bioceramics demonstrated a good mechanical property, the printed bioceramics typically need to be post-processed, such as being cured or sintered after the 3D printing process, which is time-consuming and can degrade the biological properties. The HA/polyethylene composites with the addition of HAWs exhibited a higher mechanical property than that of spherical HA. The optimized PLLA (L-polylactic acid) composites with the addition of nHA were fabricated by fused deposition modeling, which can be applied to clinical applications [21]. The elastic modulus of 30% nHA composites and 50% nHA composites was 45.54 ± 0.11 MPa and 44.31 ± 0.10 MPa, respectively. The 50% nHA composite specimen showed brittleness. By integrating nHA and PLGA using a table-top stereolithography 3D printer, biomimetic osteochondral scaffolds had hierarchical nano-to-micro structure and spatiotemporal bioactive factor gradients [22]. Ultra-high molecular weight polyethylene composites with HA as filler were fabricated by fused deposition modeling, showing that the inclusion of 50 wt% HA can reduce the degradation temperature in TGA and DSC and improve the processability in the printing process [23]. The mechanical properties of polylactic acid can be improved with the addition of different levels of HA content (5 and 15 wt%) [24]. However, the interface bonding of composites was not good enough to improve the mechanical properties of the whole scaffold. Interface bonding is related to the uniformity, strength, and functionality of composite scaffolds. As well, plasticizers have become a very important additive in polymer systems to modify polymers [25]. Honary et al. [26] used the plasticizers to improve the mechanical and conditional quality of hydroxypropyl methyl cellulose (HPMC) films, and the thermomechanical properties were optimized by different grades of plasticizers. Hence, the addition of different polymers or the modified polymers with plasticizers in a hydroxyapatite scaffold will have a positive impact on its printability and mechanical property.

In addition, a HAW-reinforced polymer scaffold has poor biodegradability or its degradation products could cause some side effects in the surrounding tissue due to the high content of polymers. The study on the hydroxyapatite/poly-lactic-co-glycolic acid (HA/PLGA) composite demonstrated that the nano HA was more uniformly dispersed in the PLGA matrix without any cavities, which was able to promote the crystallization effectiveness of the HA/PLGA composite compared with HAWs [27]. The degradation of the PLGA could be accelerated with the addition of nano HA when nano HA was detached from the PLGA during the degradation [28]. As well, Huang et al. [29] investigated three polymeric composite scaffolds based on the quickly degradable poly-lactic-co-glycolic acid (PLGA), the slowly degradable polycaprolactone (PCL), and the non-degradable polyamide 66 (PA66), which showed that the nHA/PCL scaffold degraded slowly after 6 months (−20% degradation) and the nHA/PA66 scaffold showed no degradation during the entire 12 months. Therefore, in view of the slow degradation of polymers, it is necessary to introduce bioceramics to accelerate their degradability. However, there are few reports on the influence of HA whiskers on the microstructure, phase, and mechanical property of bioceramic scaffolds. In the study, HA whiskers (HAWs) with properly sized reinforced HA scaffolds were directly prepared by 3D printing without post-processing. The effects of polyvinyl alcohol (PVA) and HPMC on the 3D printing of the HA scaffolds were studied. The effects of HA whisker content and the additives on the microstructure, phase, and mechanical property of the scaffolds were investigated in detail. The biomimetic HA whisker-reinforced HA (HAW/HA) scaffold, with similar mechanical property and inorganic components to those of human bone, was printed and could be used in various bone defect sites. The study also offered a 3D printing process without post-processing to prepare a ceramic scaffold with a high ceramic content (>80%) and excellent mechanical property, which was a serious challenge because of the difficulty of forming and printing, insufficient cohesion, and low mechanical property of the scaffold.

## 2. Materials and Methods

### 2.1. Reagents and Materials

Calcium nitrate tetrahydrate (Ca (NO_3_)_2_·4H_2_O, AR), acetamide (AA, C_2_H_5_NO, AR), diammonium hydrogen phosphate ((NH_4_)_2_HPO_4_, AR), nitric acid (HNO_3_, AR), and ammonia water (NH_3_·H_2_O, AR) were used to synthesize HAWs. Spherical HA (Ca_10_(PO_4_)_6_(OH)_2_) was procured from Shanghai Aladdin Biochemical Technology Co., Ltd. (Shanghai, China). Glycerol (C_3_H_8_O_3_), HPMC (C_20_H_38_O_11_), n-octanol (C_8_H_18_O), and PVA ([-CH_2_CHOH-]_n_) were provided by Sinopharm Chemical Reagent Co., Ltd. (Shanghai, China). Polyacrylamide (PAA-NH_4_) was obtained from Macklin Chemical Reagent Co., Ltd. (Shanghai, China). HAWs with a high aspect ratio were synthesized in a solution consisting of calcium and phosphorus ions with an atomic Ca/P ratio of 1.67 by a hydrothermal synthesis method [30,31]. In this study, 84 mmol/L Ca(NO_3_)_2_ solutions, 1.25 mol/L C_2_H_5_NO (AA) solutions, and 50.3 mmol/L (NH_4_)_2_HPO_4_ solutions were mixed at a ratio of 1:1:1. The pH of the mixed solution was adjusted to 4.5 by adding dilute NH_3_·H_2_O solution for the preparation of HAWs.

### 2.2. Fabrication of HAW/HA Scaffolds

HAWs and spherical HA powder with mass ratios of 1:9, 2:8, 3:7, 4:6, and 5:5 were added to 1.5 wt% HPMC aqueous solution at a mass ratio of 1:1 and were then uniformly mixed, followed by centrifugation at 2200 rpm for 15 min and vacuum pumping at 85 KPa for 2 min. The centrifugation and vacuum pumping were repeated 3 times to remove bubbles, and HAW/HA ink was obtained. The HAW/HA ink was loaded into a storage barrel which connected with a screw extruder and an air pump and was then printed through a conical nozzle at a speed of 1.0 mL/min. The 3D printer nozzle with a printing head diameter of 1200 μm was used to print the scaffold at room temperature. The printing spacing and layer spacing were set at 800 μm. The HAW/HA ink was extruded from the nozzle under the action of a screw extruder and deposited on a work platform by a layer-by-layer printing method with a printing speed of 50 mm/min. The dimension of the HAW/HA scaffold was 10 × 10 × 12 mm^3^. After printing, the printed scaffold was dried at 40 °C for 24 h. The HAW/HA scaffolds were reinforced by whiskers with a HAW/HA ratio of 0:10, 1:9, 2:8, 3:7, 4:6, and 5:5 and were named the 0-HAW/HA, 1-HAW/HA, 2-HAW/HA, 3-HAW/HA, 4-HAW/HA, and 5-HAW/HA scaffold, respectively.

When PVA was used in the 3D printing, it was dissolved in DI water for 4 h, heated to 60 °C, and kept for 4 h; then, it was held at 98 °C for 4 h to obtain a 10 wt% PVA aqueous solution [32,33]. HAWs and HA powder with a ratio of 4:6 were added to the aqueous solution of PVA at a mass ratio of 1:1 and mixed for 30 min to obtain a HAW/HA ink. The HAW/HA scaffolds reinforced with whiskers were fabricated using the HAW/HA ink and were denoted as 4-HAW/HA-PVA.

### 2.3. Characterization

Surface and cross-sectional morphologies of the HAW and HAW/HA scaffolds were observed by scanning electron microscopy (SEM, FEI Verios 460 instrument, Hillsboro, OR, USA). The surface morphology and roughness of the HAW/HA scaffolds were observed under an ultra-depth-of-field microscope (KH-8700, Hirox, Tokyo, Japan). X-ray diffraction (XRD) was used to analyze the phase of the HA powder and HAW/HA scaffolds by a Smart Lab X-ray diffractometer with a voltage of 9 kV (AD/max2200/PC, Rigaku, Tokyo, Japan) at 5~80°. Fourier transform infrared spectroscopy (FTIR) was performed by using a FTIR spectrometer (VERTEX-80, Bruker, Hamburg, Germany). The rheological property of the HAW/HA ink was measured at 25 °C using an ARES rheometer (DHR-1, TA instruments, Newark, DE, USA). The porosity of the HAW/HA scaffolds was evaluated by the Archimedes method.

The compressive strength of the HAW/HA scaffolds (sample size 5 × 5 × 5 mm^3^) was conducted using a Universal Testing Machine (XWW-20B, Chengde Kecheng Testing Machine Co., Ltd., Chengde, China) with a load cell of 10 kN at 0.5 mm/min [34]. The compressive strength and compressive strain of the HAW/HA scaffolds were evaluated using three specimens for each group to obtain an average value. Compressive strength (*σ_c_*) and compressive strain (*ε*) were calculated according to Equations (1) and (2), respectively.

(1) $$\sigma_{c}=\frac{p}{s}$$

where *σ_c_* represents the compressive strength (MPa), *p* represents the maximum load of the sample under compression (N), and *s* indicates the area of the sample pressure surface (mm^2^).

(2) $$\varepsilon =\frac{∆L}{L}$$

where *ε* represents the compressive strain (mm/mm), Δ*L* represents the variation of specimen length, and *L* indicates the original length of the specimens.

### 2.4. Statistical Analysis

The statistical significance of the compressive strength between any two groups was assessed by independent sample *t*-test. A *p* value of less than 0.05 or less than 0.01 was marked as statistically significant or as highly significant, respectively.

## 3. Results and Discussion

### 3.1. Morphologies of HA Whiskers

**Figure 1:**
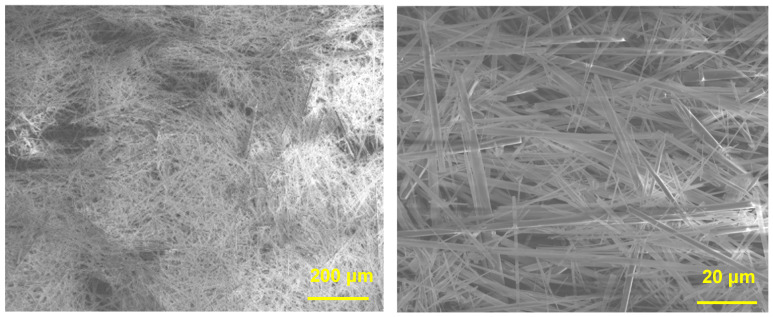
SEM images of HAWs.

The 3D printing of a ceramic scaffold with a high ceramic content (>80%) is a serious challenge because of the difficulty of forming and printing, insufficient cohesion, and low mechanical property of the scaffold. In the study, HAWs with a suitable aspect ratio and content were able to improve cohesion in the HA scaffolds, which allowed the bioceramic content in the scaffolds to reach 83%. The microscopic morphology of HAWs is shown in Figure 1. The length of a strip-like HAW was 100–200 μm, the diameter was 1–3 μm, and the aspect ratio was about 70–100. HAWs with a high aspect ratio can be used as a suitable reinforcement and can greatly enhance the mechanical properties and structural stability of composites. Strong and biocompatible HAWs were synthesized to reinforce the HA scaffolds, which were then able to possess an improved toughness without degradation of biocompatibility [35].

**Figure 2:**
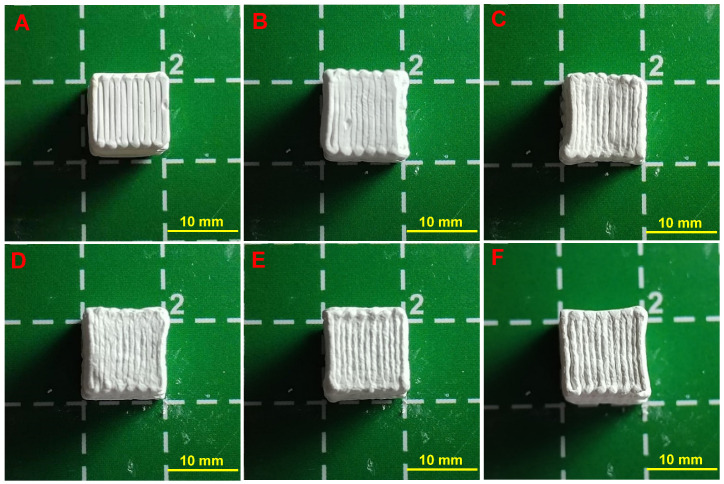
Morphology of (**A**) 0-HAW/HA, (**B**) 1-HAW/HA, (**C**) 2-HAW/HA, (**D**) 3-HAW/HA, (**E**) 4-HAW/HA, and (**F**) 5-HAW/HA.

**Figure 3:**
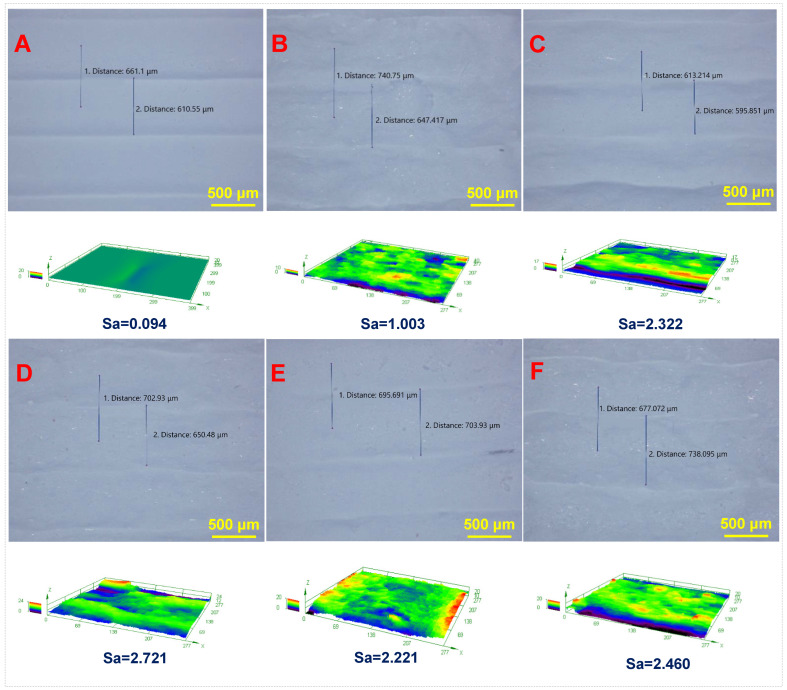
Surface morphology and roughness of (**A**) 0-HAW/HA, (**B**) 1-HAW/HA, (**C**) 2-HAW/HA, (**D**) 3-HAW/HA, (**E**) 4-HAW/HA, and (**F**) 5-HAW/HA.

The macroscopic morphology of the HAW/HA scaffolds is presented in Figure 2. It can be seen that the surface of the HAW/HA scaffolds became rough with the increases in HAW content. The surface morphology and roughness of the HAW/HA scaffolds with different ratios (0-HAW/HA, 1-HAW/HA, 2-HAW/HA, 3-HAW/HA, 4-HAW/HA, and 5-HAW/HA) observed under an ultra-depth-of-field microscope are shown in Figure 3A,F. The surface of the 0-HAW/HA scaffold without HA whiskers was relatively smooth, and the surface roughness parameter Sa was 0.094. However, the surface roughness and morphology of the scaffolds were affected when the HA whiskers were introduced. The surface roughness of the 1-HAW/HA scaffold increased with an increasing HA whisker content. Some bright spots could be observed on the surface of the scaffold with a HAW/HA ratio of 1:9 (1-HAW/HA). As shown in Figure 3D, more bright spots in the HAW/HA scaffolds could be seen when the HAW/HA ratio increased from 2:8 to 3:7. The surface roughness parameter Sa was 2.221. The roughness of the HAW/HA scaffolds increased with the addition of the HAWs, which may have been caused by the larger grain size of the HAWs. The roughness of the HAW/HA scaffolds (3-HAW/HA, 4-HAW/HA, and 5-HAW/HA) did not obviously change compared with that of the 2-HAW/HA scaffold (Figure 3E,F). The surface roughness and morphology of biomaterials are crucial factors that influence cell adhesion [36,37]. A greater surface roughness and a more irregular surface morphology would be to the benefit of cell adhesion [38]. A surface roughness of between 1 and 2 μm is beneficial to cytocompatibility and cell adhesion [39]. Therefore, the increased surface roughness of the HAW/HA scaffolds could be beneficial for cell adhesion and proliferation during bone repair [40,41]. In addition, the porosity of the 4-HAW/HA scaffold was 77%, which would help improve cell adhesion, proliferation, migration, and differentiation [42].

**Figure 4:**
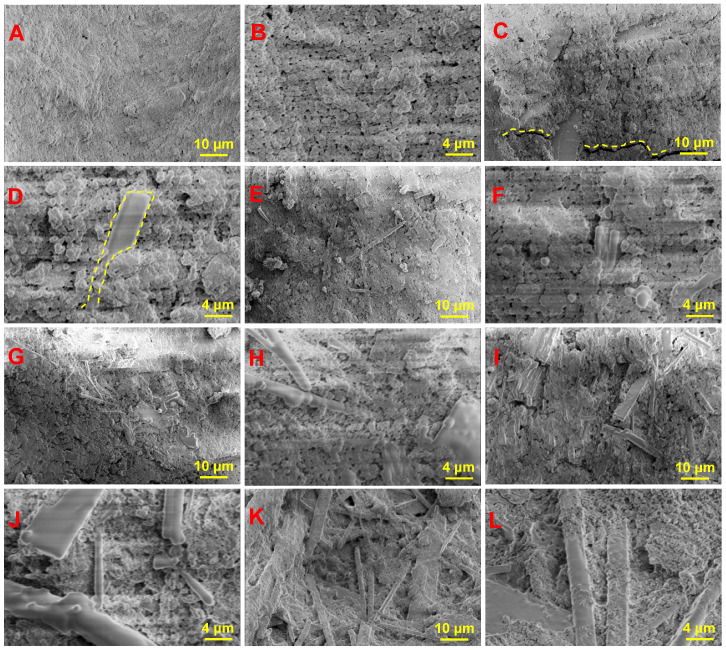
SEM images of (**A**,**B**) 0-HAW/HA, (**C**,**D**) 1-HAW/HA, (**E**,**F**) 2-HAW/HA, (**G**,**H**) 3-HAW/HA, (**I**,**J**) 4-HAW/HA, and (**K**,**L**) 5-HAW/HA.

The microscopic morphologies of 0-HAW/HA, 1-HAW/HA, 2-HAW/HA, 3-HAW/HA, 4-HAW/HA, and 5-HAW/HA are presented in Figure 4. The surface of 0-HAW/HA was smooth and flat, and the spherical HA grains were tightly bonded. There were many irregular pores on the surface. As shown in Figure 4C,L, with increasing HAW content, the surface roughness of the HAW/HA scaffolds increased. When the HAW/HA ratio was 1:9 in the scaffold, a few strip-like HAWs appeared in the 1-HAW/HA scaffold, as shown in Figure 4C. More HA whiskers could be observed in the HAW/HA scaffolds with the increase in HAW content. When the HAW/HA ratio reached 4:6, it could be observed that HAWs were uniformly distributed in the HAW/HA scaffold, as shown in Figure 4I,J. HAWs filled the pores formed in the HA matrix and created a bridge effect in the scaffolds, which could have a positive impact on the mechanical properties of the scaffolds [43,44]. When the HAW/HA ratio was increased to 5:5, some HAW agglomeration could be observed with this HAW/HA ratio increment, which could be detrimental to the mechanical properties of the HAW/HA scaffolds [45].

### 3.2. Mechanical Properties of HAW/HA Scaffolds

**Figure 5:**
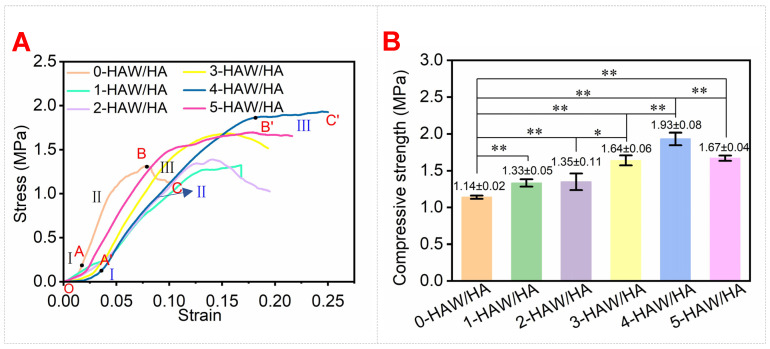
(**A**) Compressive stress–strain curve of HAW/HA scaffolds and (**B**) compressive strength of HAW/HA scaffolds with different HAW content. (* *p* < 0.05, ** *p* < 0.01).

Figure 5A presents the compressive stress–strain curves of the HAW/HA scaffolds with different HAW ratios. The compressive stress–strain curves of the HAW/HA scaffolds were similar to the previously reported results [46]. The stress–strain curve of the 0-HAW/HA scaffold demonstrated a typical brittle fracture behavior and was almost linear when the strain was below 0.075. The fracture strain was less than 0.10 when the stress reached the fracture strength. In comparison, the fracture strain and strength of the HAW/HA scaffolds with HAWs increased, which indicated that the deformability and compressive strength of the HAW/HA scaffolds improved with the addition of the HAWs. The enhancement of the compressive strength of the HAW/HA scaffolds can be attributed to the strengthening effect. The energy absorption capacity can be calculated by the area under the compressive stress–strain curves up to a predetermined strain, which is thought to be a form of toughness [47,48]. The energy absorption capacity of the 0-HAW/HA, 1-HAW/HA, 2-HAW/HA, 3-HAW/HA, 4-HAW/HA, and 5-HAW/HA scaffolds was 0.088, 0.157, 0.162, 0.205, 0.301, and 0.261 MJ/m^2^, respectively. The 4-HAW/HA scaffold demonstrated the highest energy absorption capacity among the HAW/HA scaffolds, which indicated that the addition of HAWs with a suitable ratio (HAW/HA ratio of 4:6) had an obvious strengthening and toughening effect on the HAW/HA scaffolds because of the more uniform distribution of HAWs in the 4-HAW/HA scaffold. As shown in Figure 5A, the compressive stress–strain curve of the 0-HAW/HA and 4-HAW/HA scaffold included three stages. Stage Ⅰ (OA and OA′) in the stress–strain curve showed linear elastic deformation. In stage Ⅱ (AB and A′B′), the stress increased rapidly, and the scaffold became denser with the increasing stress. In stage Ⅲ (BC) of the 0-HAW/HA scaffold, the scaffold collapsed completely [34]. Stage Ⅲ (B′C′) of the 4-HAW/HA scaffold did not demonstrate a typical brittle failure. Moreover, greater compressive stress was generated, and fracture failure was delayed, both due to the strengthening and toughening effect of uniformly distributed HAWs, which resulted in higher strength and toughness of the 4-HAW/HA scaffold.

As shown in Figure 5B, the compressive strength of the HAW/HA scaffolds with different HAW content significantly increased compared with that of the HA scaffold (** *p* < 0.01). The compressive strength of the HAW/HA scaffolds was enhanced significantly when the HAW/HA ratio was increased from 2:8 to 4:6, and this strength was reduced when the HAW/HA ratio reached 5:5. The compressive strength of the 0-HAW/HA scaffold was 1.14 ± 0.02 MPa. With the addition of HA whiskers (HAW/HA ratio of 1:9), the compressive strength of the HAW/HA scaffold was enhanced to 1.33 ± 0.05 MPa. When the HAW/HA ratio was increased to 4:6, the compressive strength of the 4-HAW/HA scaffold became 1.93 ± 0.08 MPa and was 69.3% higher than that of the 0-HAW/HA. The maximum strain was approximately 2.5 times that of the 0-HAW/HA. The compressive strength of the HAW/HA scaffolds with 0-HAW/HA, 1-HAW/HA, 2-HAW/HA, 3-HAW/HA, 4-HAW/HA, and 5-HAW/HA was 1.14 ± 0.02, 1.33 ± 0.05, 1.35 ± 0.11, 1.64 ± 0.06, 1.93 ± 0.08, and 1.67 ± 0.04, respectively. However, the compressive strength of the 5-HAW/HA scaffold significantly decreased when the HAW/HA ratio was increased from 4:6 to 5:5. This may be due to the fact that the agglomeration of HAWs lowered the compressive strength of the HAW/HA scaffolds.

**Figure 6:**
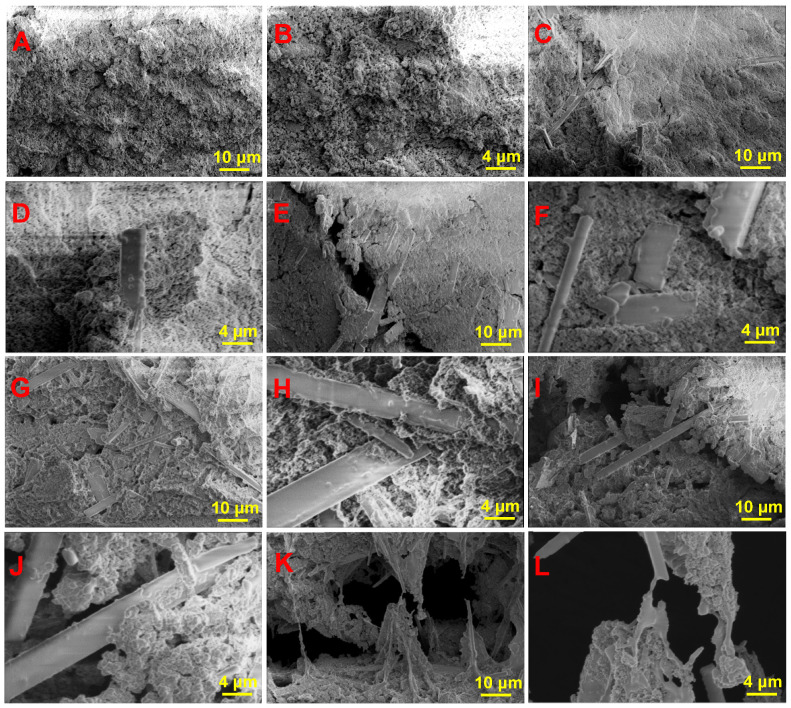
Cross-sectional morphologies of (**A**,**B**) 0-HAW/HA, (**C**,**D**) 1-HAW/HA, (**E**,**F**) 2-HAW/HA, (**G**,**H**) 3-HAW/HA, (**I**,**J**) 4-HAW/HA, and (**K**,**L**) 5-HAW/HA.

Cross-sectional morphologies of the HAW/HA scaffolds are shown in Figure 6. The HA scaffolds without HAWs displayed a flat cross section, which was an obvious brittle fracture (Figure 6A,B). Some strip-like HA whiskers could be observed on the fracture surface of 1-HAW/HA (Figure 6C,D). There were some cracks on the fracture surface of the 2-HAW/HA scaffold with a HAW/HA ratio of 2:8 (Figure 6E). The whiskers bridging in the cracks can be clearly observed. When this scaffold was subjected to stress, there was partial debonding of the HAWs along the crack, leading to a bridging between the whiskers [49]. A continuous extension of microcracks would be inhibited with the addition of HAWs, and more fracture energy would be consumed during the process. When the HAW/HA ratio was 3:7 in the scaffold, the fracture surface of the 3-HAW/HA scaffold became rougher, as shown in Figure 6G,H. As the HAW/HA ratio reached 4:6, more whisker pullout can be observed, as shown in Figure 6I,J. A portion of the energy was also dissipated by whisker pullout and breakage, thereby hindering crack propagation and retarding the rate of crack propagation [50]. HAW pullout as a reinforcement could be beneficial in improving the ability of HAW/HA scaffolds to withstand loads [51]. Moreover, these elongated HAWs impeded crack propagation and increased the strength of HAW/HA scaffolds. Furthermore, their presence promoted crack deflection, which could disperse stress concentration and prevent catastrophic failure [52]. However, when the HAW/HA ratio was further increased, not only the agglomeration of HAWs but also whisker pullout could be observed, likely resulting in a reduction of the compressive strength of the HAW/HA scaffolds.

### 3.3. Rheological Property of HAW/HA Ink

**Figure 7:**
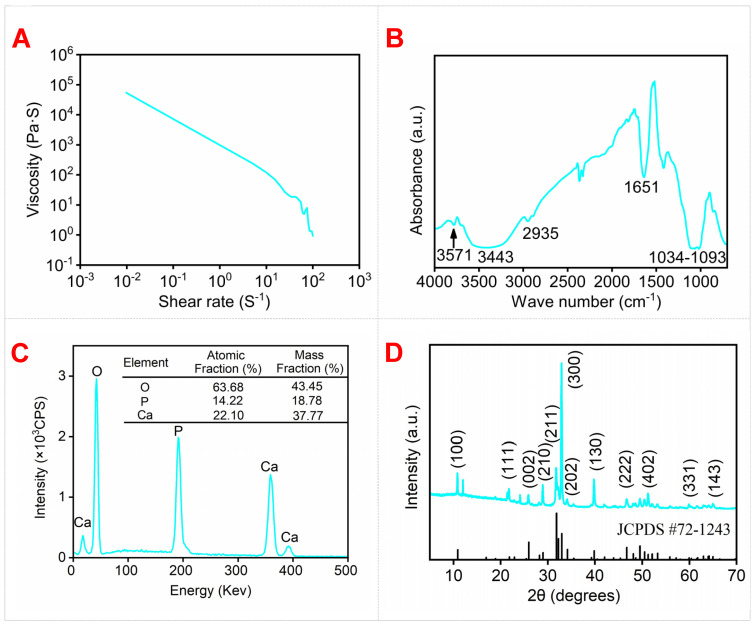
(**A**) Rheological property of 4-HAW/HA ink, (**B**) FTIR, (**C**) EDS, and (**D**) XRD pattern of 4-HAW/HA scaffold. Vertical lines represent HA standards according to JCPDS # 72-1243.

Taking into account the excellent mechanical properties of the 4-HAW/HA scaffold, rheological analysis, FTIR, EDS, and XRD were further performed. The rheological properties of the 4-HAW/HA ink had a great effect on the stability of the 3D printing [53]. The shear-thinning property of an ink is an essential precondition for the 3D printing process. The ink can easily flow through the printing nozzle under the shear action of the screw at a shear rate (≥−10 s^−1^). As shown in Figure 7A, the viscosity of the ink decreased as the shear rate increased. The FTIR spectrum for 4-HAW/HA (Figure 7B) shows characteristic bands of the phosphate group at 1093 and 1034 cm^−1^. The band for hydroxyl at 3571 cm^−1^ was a characteristic band of HA [54]. The characteristic phosphate group band of HPMC at 3443 cm^−1^ was related to the -OH bond, at 2935 cm^−1^ caused by the C-H bond, and at 1651 cm^−1^ and 1069 cm^−1^ caused by C=O and C-O-C bond, respectively [55]. This indicated the presence of HPMC in the scaffold. The EDS spectra of 4-HAW/HA are shown in Figure 7C. The 4-HAW/HA scaffold contained 22.10% Ca, 14.22% P, and 63.68% O elements (atomic fraction), and the mass fraction of Ca, P, and O elements was 37.77%, 18.78%, and 43.45%, respectively. The Ca/*p* atomic ratio was 1.55. As shown in Figure 7D, typical diffraction peaks of 4-HAW/HA corresponded to the crystal planes of HA (JCPDS # 72-1243).

### 3.4. Microscopic Morphology and Performance of 4-HAW/HA-PVA

**Figure 8:**
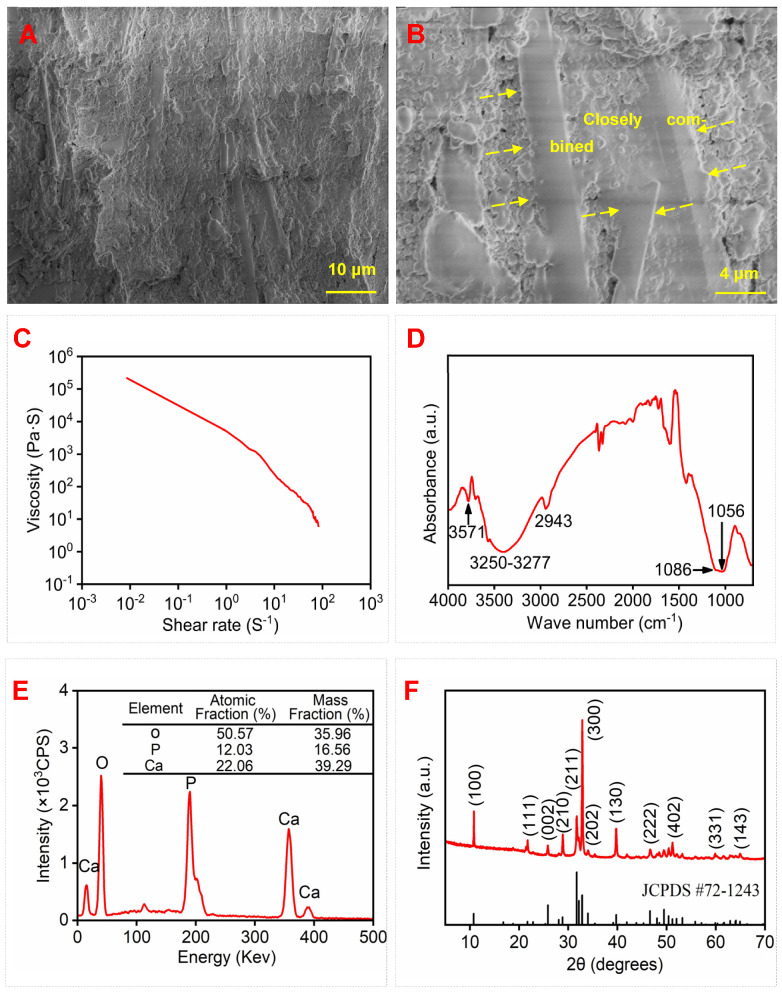
(**A**,**B**) SEM images, (**C**) rheological property, (**D**) FTIR, (**E**) EDS, and (**F**) XRD pattern of 4-HAW/HA-PVA scaffold. The yellow arrows represents the bonding location of PVA, HAWS and spherical HA. Vertical lines represent HA standards according to JCPDS # 72-1243.

Biocompatible polyvinyl alcohol (PVA) is widely used as artificial cartilage and meniscus and is used in tissue adhesion barriers [56]. When PVA was used as an additive in the scaffold, the surface of 4-HAW/HA-PVA was smoother and a good bonding was formed between PVA, HAWs, and spherical HA compared with the 4-HAW/HA scaffold, as shown in Figure 8A,B.

**Figure 9:**
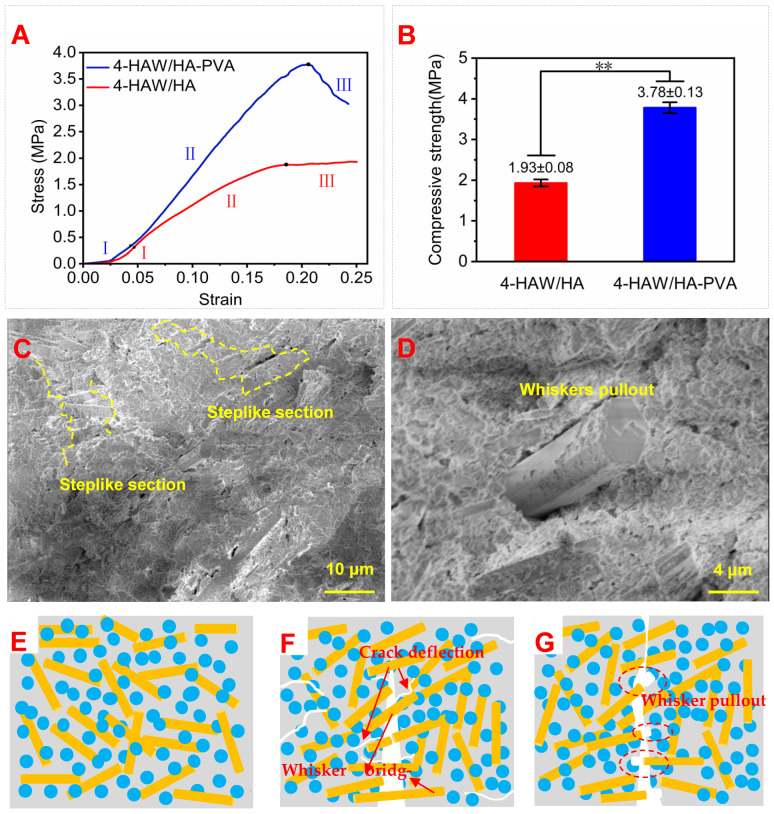
(**A**) Compressive stress–strain curves, (**B**) compressive strength of 4-HAW/HA and 4-HAW/HA-PVA scaffolds (** *p* < 0.01), (**C**,**D**) cross-sectional morphologies of 4 -HAW/HA-PVA, (**E**) the reinforcing toughing mechanism of PVA and hydroxyapatite bonding, (**F**) crack deflection and whisker bridging, and (**G**) whisker pullout. In the figure (**E**–**G**), the gray represents the 4 -HAW/HA-PVA matrix, the blue dot represents spherical HA, the yellow bars represents HA whisker.

This smoothness and the good bonding can greatly improve the mechanical property of the scaffold [57]. Rheological analysis, FTIR, EDS, and XRD were performed to study the rheological property, functional groups, composition, and phase of 4-HAW/HA-PVA. The 4-HAW/HA-PVA ink (Figure 8C) presented a rheological property similar to that of the 4-HAW/HA ink (Figure 7A). As shown in Figure 7A and Figure 8C, the viscosity of 4-HAW/HA ink and 4-HAW/HA-PVA ink decreased rapidly with the increase of shear rate when the shear rate was greater than 10^−2^ S^−1^, exhibiting an obvious shear-thinning phenomenon. The ink for printing the 4-HAW/HA-PVA scaffold demonstrates a pseudoplastic rheologic behavior. The viscosity of the 4-HAW/HA-PVA ink was higher than that of 4-HAW/HA, possibly due to more hydroxyl (-OH) groups in PVA that can form strong hydrogen bonds to enhance intermolecular interaction and increase liquid viscosity [58,59]. In contrast, although HPMC also had hydroxyl groups, the ink viscosity of 4-HAW/HA ink was low because the rigidity and arrangement of its chains could lead to the formation and rupture of hydrogen bonds, which indicated that the flow properties of the inks consisting of PVA and HPMC were suitable for 3D printing. As shown in Figure 8D, the characteristic bands of the 4-HAW/HA-PVA scaffold were attributed to the characteristic bands of -OH at 3277 cm^−1^, symmetric -CH_2_ at 2943 cm^−1^, and C-O at 1086 cm^−1^ in PVA [60,61]. The EDS result revealed that the scaffold was composed of Ca, P, and O elements, and the Ca/P in the 4-HAW/HA-PVA scaffold was 1.83. The scaffold contained 22.06% Ca, 12.03% P, and 50.57% O elements (atomic fraction), and the mass fraction of Ca, P, and O elements was 39.29%, 16.56%, and 35.96%, respectively. The XRD pattern of the 4-HAW/HA-PVA scaffold is displayed in Figure 8F. The characteristic diffraction peaks of 4-HAW/HA-PVA corresponded to the crystal planes of HA (JCPDS # 72-1243). As shown in Figure 7D and Figure 8F, for the XRD pattern of the 4-HAW/HA scaffold and the 4-HAW/HA-PVA scaffold, the characteristic diffraction peaks (100), (211), (300), and (130) of HA can be seen. In addition, the peaks at 32.86°, 39.75°, 46.66°, 52.03°, 60.35°, and 66.36° corresponded to the (300), (130), (222), (402), (331), and (143) crystal planes of HA. The diffraction peaks were ascribed to HA, and other impurities did not occur in the scaffolds, which indicated that the composition and phase can hardly be changed with the addition of HPMC and PVA. Moreover, he XRD peaks of the scaffolds were not obviously broadened and were shifted along the 2θ axis, which may be because the scaffolds were printed at room temperature and contained a small amount of polymers. Compressive stress–strain curves and compressive strength of the scaffolds are demonstrated in Figure 9A,B. As shown in Figure 9A, the compressive stress–strain curve of the 4-HAW/HA-PVA scaffold included linear elastic deformation, rapid stress increase, and failure stages. Compared with the compressive stress–strain curve of the 4-HAW/HA scaffold, the curve of the 4-HAW/HA-PVA scaffold bore a higher stress at the same strain. The energy absorption capacity of the 4-HAW/HA-PVA scaffold was 0.489 MJ/m^2^, which was 1.63 times that of the 4-HAW/HA scaffold (0.301 MJ/m^2^), which suggested that the 4-HAW/HA-PVA scaffold had a better energy absorption capacity than the 4-HAW/HA scaffold. The compressive strength of the 4-HAW/HA-PVA scaffold was 3.78 ± 0.13MPa, which was 95.9% higher than the 4-HAW/HA scaffold (Figure 9B). It is notable that the compressive strength of the 4-HAW/HA-PVA scaffold was similar to that of cancellous bone (2–12 MPa), allowing the 4-HAW/HA-PVA scaffold to be applied to bone defect repair [62,63]. As shown in Figure 9C,D, obvious step-like sections occurred on the 4-HAW/HA-PVA scaffold, which was caused by debonding and slipping between the whiskers and the matrix. As shown in Figure 9E, the good bond between PVA and HA contributed to the enhancement of the comprehensive strength of the 4-HAW/HA-PVA scaffold. Whiskers were uniformly dispersed in the spherical HA and PVA. Crack deflection and HAW bridging and pullout consumed more energy (Figure 9F,G). In addition to the reinforcing mechanisms, including crack deflection, crack bridging, and whisker pullout mentioned previously in the scaffold with HPMC, the generated step-like sections consumed more energy during the fracture process, and the fracture toughness of the scaffold was improved.

## 4. Conclusions

Biomimetic HAW-reinforced HA scaffolds were prepared through 3D printing without post-processing. The presence of HAWs and the additive exhibited great effects on the morphology and mechanical property of the HAW/HA scaffold. Bioceramic content (HAWs and spherical HA) in the scaffold reached 83%, and the addition of whiskers with a HAW/HA ratio of 4:6 in the scaffold demonstrated a better strengthening and toughening effect on the HAW/HA scaffolds. The compressive strength of the 4-HAW/HA scaffold was 1.93 ± 0.08 MPa, which was 69.3% higher than 0-HAW/HA, and the maximum strain was approximately 2.5 times that of 0-HA/HA. The energy absorption capacity of the 4-HAW/HA scaffold was 0.301 MJ/m^2^, which was the highest energy absorption capacity among the HAW/HA scaffolds. When PVA was used as an additive in the scaffold, the surface of the 4-HAW/HA-PVA scaffold was smoother and a good bonding was formed between PVA and hydroxyapatite compared with the 4 -HAW/HA scaffold. The compressive stress–strain curve of the 4-HAW/HA-PVA scaffold included linear elastic deformation, rapid stress increase, and failure stages. Compared with the 4-HAW/HA scaffold, the 4-HAW/HA-PVA scaffold bore a higher stress at the same strain. The energy absorption capacity of the 4-HAW/HA-PVA scaffold was 0.489 MJ/m^2^, which was 1.63 times that of the 4-HAW/HA scaffold (0.301 MJ/m^2^), suggesting an improved toughness of the scaffold. The compressive strength of the 4-HAW/HA-PVA scaffold was 3.78 ± 0.13 MPa, which was 95.9% higher than that of the 4-HAW/HA scaffold. The good bonding between PVA and hydroxyapatite contributed to the enhancement of the strength of the scaffold. The reinforcing mechanism included crack deflection, whisker bridging, whisker pullout, and the generated step-like sections that could consume more energy during the fracture process. The mechanical property and inorganic component of the biomimetic HAW/HA scaffold were similar to those of human bone, which made it ideal for repairing bone defects. The study also offers a 3D printing process without post-processing to prepare a ceramic scaffold with higher ceramic content (>80%) and excellent mechanical property, which is a serious challenge because of the difficulty of forming and printing, insufficient cohesion, and low mechanical property of the scaffold.

## Data Availability

The original contributions presented in the study are included in the article; further inquiries can be directed to the corresponding author.
